# A Novelette

**Published:** 1915-03

**Authors:** R. Ottolengui


### A NOVELETTE.

BY R. OTTOLENGUI, M. D. S., D. D. S.

I was dining alone one night recently at the *Café des Enfants,* as we call it in the higher circles of society; but as some of you may not be in our set, let me explain that the common people know the place as Child's Restaurant. I was dining there because I was dining alone. When I have company, of course, I am compelled to eat at the Biltmore, the Ritz Carleton or the *Café des Beaux Arts;* places where the waiter does not ask: "Will you have wine, Doctor?" but pleasantly accosts you with: "Doctor, which wine will you have?" So when I am eating alone, I find it economical to patronize one of the Child's (not Children's) Restaurants, where the worst that the waiter dares to say is: "Coffee, with or without milk?" Milk, mind you, not cream!

To get back to my story, I was dining alone at Child's, or rather I was expecting to dine alone, but the Fates willed it otherwise. Did you ever approach a curb on a dark and rainy night? You naturally expect a lot of water in the drain right by the curb; so, to avoid wetting your feet, you take a long, long step, and you step completely over the dry place, and land ankle deep in a puddle? You have had the experience? Yes? I thought so. Well, that is how it was. I really passed right by the Knickerbocker, where they have a fine Grill, with good food, and where I have a favorite waiter; one you know who actually says "Thanks" when you give him a tip, and otherwise treats you like a human being, rather than just a common customer. As I was saying, I went right by the Knickerbocker, and went on and into Child's Restaurant, just hoping to have a quiet, lonesome little meal, with no chance of meeting any millionaire dentists who would insist on talking dentistry, or what they think is dentistry, when lo! And listen! What do you suppose? But let me tell you.

I had just received my two soft boiled eggs, and after removing the outer and inner peel, I was utilizing my strong right arm to mash the edible parts sufficiently so that I could mix a little butter with the yolks and whites commingled as it were, or as they were not, but as every properly cooked soft boiled egg ought to commingle, when without the slightest attempt at eaves-dropping on my part, there floated to my ears across the fan-cooled atmosphere of that restaurant these sonorous words:

"The trouble with the editor of *'Items of Interest'* is that he thinks he knows it all!"

Now, Honest! Folks, Cross my heart! Hope to die! That is not true. There are just oodles of things I don't know, and one thing that I do know, is that I don't know oodles of things. Now, just for instance, I don't know who made the war. But that isn't such crass ignorance, because very few people do know. But be that as it may, there are also loads of things right in and about dentistry, that I do not know, and would be glad to know.

Of course I did not want to listen to that conversation, because you know what the book says that listeners always hear about themselves. Still, I must confess that I opened both ears and did listen. And it proceeded thuswise.

"Oh! I would not say that exactly." This from Man No. Two. "I guess he knows a few things at that." "Well I guess that you miss your guess," said Man No. One. "For instance what does he know about mechanical dentistry? Have you seen anything about mechanical dentistry in that Around the Table talk. Yes, you have, Not."

Don't you see the situation? Had hoped to dine alone, but could I do it after that? Hardly. So I took up my egg, my slice of bread, and my napkin, and moved over to the other table. You can do that at Child's, when you couldn't at Delmonico's without first asking the waiter's permission. But at Child's we are very Democratic, or Cosmopolitan, whichever it is. So as I say, I just moved over, and I broke right in on that conversation.

"I am no Sherlock Holmes," said I, "but I rather opine (opine is good, what?), I say I rather opine, that you gentlemen are interested in mechanical dentistry. Is there any little problem therein that I might elucidate for you?" "And who might you be," asked Man No. One. "Well," said I, "I might be the Queen of the Movies, but I am not. I am merely the Editor of 'Items of Interest.'"

Do you know I could see right away that they suspected that I had overheard part of their talk, and Man No. Two hastened to interject: "We meant no offense by what we said." "Offense!" said I, "Why, boys, you could not offend me with a dynamite bomb. But really I used to polished rubber plates once myself, and maybe I could give you a point or two."

"If you really think so," said Man No. One, "go right on where you started. Tell us something about polishing plates; and what is the quickest way to get the plate thin. Do you use scrapers or files or sandpaper."

"Why, that is easy," said I. "You may not know it, but it is a fact that I served a sort of apprenticeship with Dr. Norman W. Kingsley, one of the greatest mechanical dentists of his day. Nowadays the same sort of a man is a prosthodontist, but he does not know any more in proportion to what he ought to know than Kingsley did. And I learned quite a few mechanical tricks from the Old Man. For example, this very question of making a plate thin, and of polishing it. The best way to make a plate thin, is to make it thin. Don't make it thick and then scrape, file or sandpaper it for an hour. If you adopt that method some day you will scrape a hole through a plate, and then you can start right in and make it over again."

"But how do you make it thin," persisted Man No. One.

"Use the thinnest of wax, in waxing up. Don't be afraid to use it too thin, because your plate will be a little thicker than your wax in any event. So have your wax thin enough and your plate when vulcanized, will need no scraping. The same is true in regard to polishing. Always polish your plate before you vulcanize it."

''But how the deuce can you do that,'' asked Man No. Two.

''Simplest thing in the world,'' said I. ''After waxing up to your satisfaction, use a blow pipe and pass the flame rapidly over the wax. This slightly melts the surface and when it congeals again, it will have a fairly good polish. When thoroughly cold, rub the surface of the wax with the ball of the thumb, and it will take a fine gloss. Then burnish tin foil over the wax and after vulcanization, and removing the actual excess of rubber, which should have been forced into gates cut in the plaster, you should be able to take your plate direct to the polishing lathe without use of file or scraper.

''But if through accident or lack of care a plate is a bit too thick, I will describe a method that was invented by Dr. J. Albert Kimball and placed on the market by him for a short time. He took some wooden mandrels, made to fit the lathe, and by revolving one in the lathe he would wrap around it, into a cone, a strip of sandpaper about an inch wide. These sandpaper cones were temporarily tied with a cord, and then removed from the mandrel and the edges smeared with glue. When the glue hardened he had cones of sandpaper which could be used on the lathe, and as the sand would wear off, a strip could be removed, exposing a fresh cutting surface. These sandpaper cones, made in different grits were very useful in the laboratory. Some manufacturer ought to market them.''

''You spoke of making the trial plate thin,'' said Man No. One. ''Don't see how you can do that and use it to take your bite?''

''I did not tell you,'' said I, ''to make your trial plate thin, nor did I tell you to use a thin wax plate for taking a bite. I told you to wax up your plate with thin wax, just before flasking. There are two ways out of your dilemma. Personally I always waxed up a thin plate, and vulcanized it without any teeth on it, before ever taking the bite. In fact I used this vulcanite plate for taking the bite. One great advantage of this method is that you discover before setting up your teeth, whether your impression and model were correct, and whether your plate will stay up. Remember that almost anyone can make a plate with a vacuum chamber in it that will adhere so tightly that the patient can not pull it down. But unfortunately a patient does not pull on his teeth when chewing. Quite to the contrary, he pushes on the plate. And to test a plate you should do the same. Make a trial plate by vulcanizing it without teeth, try this in the mouth, and then with the index finger press upon every part of the plate, and if it can be dislodged, it is worthless. If it withstands such tests it will carry the teeth without disturbing the adhesion, provided, of course, the teeth are properly occluded. An improper occlusion will trip any plate.''

''If you prefer to use a wax trial plate for taking the bite, or one made of base-plate gutta-percha, then after the teeth are set up, and the labio-buccal rim added and carved to form, this rim should be waxed fast to the model, so that the teeth can not be disturbed by the next step. Then with a sharp knife, slightly warmed in the Bunsen flame, the entire palatal portion of the wax must be cut away. After this, a new palatal portion is added using wax of the thickness, or a little thinner than you desire that the plate should be.

''Where the first and really the best method is used, after making the plate without teeth, the teeth may be attached to this plate with wax, which may then be replaced with rubber in a second vulcanization. As the plate is to be vulcanized a second time it is as well not to over-vulcanize it the first time. By this method a pretty result may be had by making the plate of black or dark red rubber, and attaching the teeth with pink, leaving a slight margin of the dark rubber above the pink along the rim, for protection of the pink, which is never so strong as the red or black. Of course, as you know, the pink rubber may be bleached and the color improved by placing it in a tumbler of alcohol covered with a piece of glass and setting the same in the sun for a few hours.''

''Can you tell me a good way to repair a broken rubber plate?'' asket Man No. Two. ''I have a great many full uppers come back to me broken in half, and after vulcanizing they break again. Then after two or three repairs the plate not only looks like a patch work quilt, but the rubber is as brittle as sealing wax.''

''Yes, I can give you a point in repairing plates,'' said I, ''which I am surprised is not more commonly utilized. I think that usually the two halves of the plate are waxed together, a model run, and then some of the vulcanite is cut away on each side of the crack, and the rest of it roughened for a short distance, after which the parts are waxed up on the model, flasked, packed and vulcanized. By this method you will note that the wax must be cleansed away from the part of the old rubber which is to be lapped by the new, and unless this is thoroughly well done the dirty surface of the old rubber does not unite well with the new. In any event it is a poor way of making a repair because the old rubber, made thin, and then lapped by the new is never very strong. Whereas there is a method by which a plate after repair may be just as strong as a new plate.''

''Tell us about it,'' said both men.

''When a full upper plate comes in broken in half, or nearly broken in two, the first step, of course, is to pour a model. Next the plate should be removed from the model and with a fine jeweler's saw the entire palatal portion should be cut away, the saw passing as near the teeth as possible. That portion which has been sawed out should then be replaced on the model, together with the parts carrying the teeth, in their proper relative positions, and again fastened together with wax. The plate should then be flasked, and when the flask is opened the palatal pieces are readily taken out and the little wax that was used easily removed with boiling water. But it is at this stage, and at a time when no more wax is needed, that a sharp clean bur is used in the engine for thoroughly cutting and dovetailing into the old rubber, which is still attached to the teeth, and right into this freshly cut surface new rubber is at once packed. Rubber dissolved in chloroform may be smeared over the surface of the old rubber, but this is not necessary. If the surface is really clean when the new rubber is packed, there will be perfect union after vulcanization. In this way an entirely new palatal portion is obtained, and solidly anchored into a thick portion of the old rubber, instead of lapping a thin joint as is commonly done.

"But if the patient could break the original plate," asked Man No. Two, "why can't he break the repaired plate, even if, as you say, it is as strong as it was before?"

"The first break might have been an accident that may not recur," said I. "But there are patients, who, as you say, continually break vulcanite plates through the center. If such a person can not be persuaded to have a gold plate, then there is still another method of overcoming the difficulty. Make a new plate as follows. After setting up the teeth with plaster, take an impression of the six front teeth, the plaster flowing over their labial surfaces, and slightly over the incisive ends. When this impression is hard, remove the teeth from the wax, and place them in the impression, of course, cleansed of all wax. Next bend a piece of iridio-platinum wire of about 16 gauge, so that it will touch the pins of all six teeth. Attach the bar to the teeth with sticky wax, remove teeth and bar together and invest in your favorite soldering investment. When hard heat up slowly, but thoroughly, and solder the bar to each pin. When cold you will have your six front teeth soldered to an iridio-platinum bar, and in proper relation to one another. The teeth may be replaced on the model in proper position and when vulcanized into the plate the bar renders it almost unbreakable through the center. A bar vulcanized in the same position, but not soldered to the teeth does not accomplish the same purpose. On the contrary it weakens rather than strengthens the plate.''

''And now boys I must be going,'' said I. ''Hereafter don't ever think that I think that I know it all, because I don't. And don't think I have told you all I know, because I haven't. I hope I shall meet you again. Good night.''

As I was paying my check at the cashier's desk, one of the ways they have of doing things at Child's I heard Man No. One remark (*soto voce* I think it was): ''I don't see that he told us so much after all!''—From the ''Round Table'' in *Items of Interest.*

